# Gene Network Analysis in Amygdala following Taste Aversion Learning in Rats

**DOI:** 10.1155/2013/739764

**Published:** 2013-05-23

**Authors:** Siva K. Panguluri, Nobuyuki Kuwabara, Nigel Cooper, Srinivas M. Tipparaju, Kevin B. Sneed, Robert F. Lundy

**Affiliations:** ^1^Department of Pharmaceutical Sciences, College of Pharmacy, University of South Florida, 12901 Bruce B Downs Boulevard MDC030, Tampa, FL 33612, USA; ^2^Anatomical Sciences and Neurobiology, School of Medicine, University of Louisville, Louisville, KY 40292, USA

## Abstract

Conditioned taste aversion (CTA) is an adaptive behavior that benefits survival of animals including humans and also serves as a powerful model to study the neural mechanisms of learning. Memory formation is a necessary component of CTA learning and involves neural processing and regulation of gene expression in the amygdala. Many studies have been focused on the identification of intracellular signaling cascades involved in CTA, but not late responsive genes underlying the long-lasting behavioral plasticity. In this study, we explored *in silico* experiments to identify persistent changes in gene expression associated with CTA in rats. We used oligonucleotide microarrays to identify 248 genes in the amygdala regulated by CTA. Pathway Studio and IPA software analyses showed that the differentially expressed genes in the amygdala fall in diverse functional categories such as behavior, psychological disorders, nervous system development and function, and cell-to-cell signaling. Conditioned taste aversion is a complex behavioral trait which involves association of visceral and taste inputs, consolidation of taste and visceral information, memory formation, retrieval of stored information, and extinction phase. *In silico* analysis of differentially expressed genes is therefore necessary to manipulate specific phase/stage of CTA to understand the molecular insight.

## 1. Introduction

Taste is a unique sensory system as it provides neural information leading to the establishment and strengthening of taste preferences via associations with postingestion signals from visceral organs. Conditional taste aversion (CTA) learning is a behavioral paradigm whereby an animal, including humans, develops an aversion to a food previously associated with visceral malaise [1]. On fundamental level, the resultant gustatory memory is used in making a decision on whether the food stuff is ingested or rejected. The identification of the genes and network pathways involved in CTA will be crucial in understanding processes necessary for the initiation and maintenance of long-term taste memory, as well as how the brain extracts meaning from the sensory stream that can promote or discourage consumption.

The amygdala is a forebrain region that has been extensively studied in the context of CTA learning and memory [2–9]. Lesion studies demonstrate a prominent role for the basolateral nucleus of the amygdala (BLA) but are not in full agreement regarding the effects of damage to the central nucleus (CeA). Some studies unveiled no effect of CeA lesions on CTA [2, 5, 10], while others have shown that CeA lesions placed prior to conditioning disrupt learning [11, 12]. Yet other studies employing transient blockade of gene expression showed that disruption of cAMP response element-binding protein (CREB) or c-fos in a region overlapping CeA impairs learning [8, 13, 14]. Similar results have been observed following transient blockade of CREB and c-fos-associated intracellular signaling cascades such as protein kinase A and C [15, 16]. Together these studies provide compelling evidence that neural processing and subsequent activation of cAMP/Ca^2+^/CREB pathways in the amygdala (both BLA and CeA) plays a critical role in CTA. Transcription factor activation during the early taste/visceral associative phase of CTA implies that the formation of long-term memory requires *de novo *protein synthesis [13]. Despite these recent advances, a significant gap in knowledge remains regarding later changes in downstream gene expression. 

Based on our study using CTA rat model of learning, we report that amygdala is an important region in the brain for taste response and behavior. Therefore in our previously published study [17] we reported genes that are differentially altered in amygdala (BLA + CeA) using rat whole genome chip, and the selected genes with desired cut-off limits were validated by using quantitative RT-PCR and Western blotting or ELISA experiments [17]. We used loss-of-gain function experiments on insulin by blocking insulin receptor centrally via its peptide antagonist and showed that blocked insulin receptor delays CTA learning [17]. The molecular level understating in the field of behavioral sciences is relatively new; therefore, as a significant step in this study we utilized previously established behavior model to understand molecular level details. In the present study we further identified and expanded the scope of the experimental data published by Panguluri et al. [17] and performed in depth bioinformatics analysis and provide the interpretation which provides complete overview of this area. Two network analysis softwares were used to identify potential pathways and cellular processes affected by selected gene sets in addition to direct and indirect interactions among the differentially expressed genes [18]. We discuss the bioinformatic tools that can be used to identify networks and pathways of interest from a large data set. All the microarray analysis data can be viewed in ArrayExpress (http://www.ebi.ac.uk/microarray-as/ae/; accession number E-MEXP-3029). 

The results obtained from this study provide detailed molecular insight into central taste processing and taste aversion learning, which may help us apply this to understand many eating-related behavioral disorders such as anorexia and bulimia. In addition, several reports suggest that changes in food taste lead to aversion which is one of the major characteristics found in cancer patients [19]. Previous studies also suggest that about 45% of cancer patients loose at least 10% of their body weight, and more than 82% of patients with chemotherapy develop taste aversion [20]. We also know that altered conditional taste aversion has been reported in diabetic models [21], and manipulation of hedonic value for high-fat diet is very important to reduce obesity. 

## 2. Materials and Methods

### 2.1. Animal Model

 All the experiments in the present investigation used adult male Sprague-Dawley rats that weighed 200–275 g at the time of CTA. All animals were individually housed in cages placed in a temperature- and humidity-controlled room with food *ad libitum*. All experiments were performed in the home cage. Experimental protocols were approved by the institutional committee for this study.

### 2.2. CTA Treatment

 Animals were divided into three different groups (sucrose/NaCl, sucrose/LiCl, and LiCl/sucrose). After 5-day acclimation to fluid restriction where drinking water from a graduated cylinder fitted with a sipper tube was available for 15 min in the morning and 1 h in the afternoon, all animals had access to 0.3 M sucrose instead of water in the morning on Day 6. Thirty minutes later, animals in the sucrose/NaCl group (*n* = 5) received an intraperitoneal (ip) injection of saline (control group), while those in the sucrose/LiCl group (*n* = 5) received ip LiCl (0.15 M, 13.3 mL/kg, contingent LiCl group). Animals in the LiCl/sucrose group (*n* = 5) received ip LiCl the day prior to sucrose intake (i.e., following morning water intake on day 5; noncontingent LiCl group). For the next two days animals had access to water for 15 min in the morning and 1 h in the afternoon. This sequence was repeated a second time followed by an intake test on day 12 where all groups had access to sucrose for 15 min in the morning in the absence of ip injections. Data were presented as mean sucrose intake (± SE) and analyzed with repeated measures ANOVA. *Post hoc* contrast analyses (LSD) were used to determine the source of statistically significant differences. *P* values <0.05 were considered statistically significant.

### 2.3. Tissue Collection

 The animals were administered a lethal dose of Nembutal (150 mg/kg) immediately following the sucrose intake test on day 12 and decapitated after termination of respiration using a laboratory guillotine. Brains were extracted under chilled (4°C) oxygenated artificial cerebrospinal fluid (in mM: 123 NaCl, 5 KCl, 1.2 KH_2_PO_4_, 15 glucose, 30 NaHCO_3_, 1.3 MgSO_4_, and 2.4 CaCl_2_) adjusted to pH 7.4, blocked for tissue containing the amygdala, rapidly frozen on dry ice, and stored at −80°C until further use. Tissue samples for RNA and protein analyses were obtained from thick cryostat cut sections (250 *μ*m). Both the central and basolateral nuclei were dissected bilaterally from six frozen sections using established anatomical landmarks (i.e., bounded dorsal by the striatum, medial by the optic tract, and lateral by the external capsule). In rodents, neural processing in visual cortex does not play a role in CTA and, thus, allowed assessment of nonspecific changes in gene expression; therefore we also collected visual cortex. The instruments and working area were sprayed with RNase Zap (Ambion/Applied Biosystems, CA, USA) to ensure an RNA-free environment. 

### 2.4. Microarray Analysis, Data Processing, Validation, and Behavioral Characterization

All the materials and methods related to whole rat genome oligonucleotide microarray gene chip (Agilent Technologies, USA), data processing, raw data analysis, validation by quantitative real-time RT-PCR, Western blot/ELISA, and behavioral characterization have been published elsewhere [17]. 

### 2.5. Network Analysis

The oligonucleotide microarray data was initially filtered by two criteria: (1) *P* value with ≤0.05 and (2) fold values (up or down) ≥2. Animals were divided into three groups with 3 animals each: (1) control group, which received sucrose intake paired with intraperitoneal (ip) saline; (2) conditioned group, which received sucrose intake paired with LiCl (0.15 M, ip); and (3) pseudoconditioned group, which received LiCl (0.15 M, ip) the day prior to sucrose intake. Briefly, animals in groups (1) and (3) do not learn an aversion to sucrose and continue to consume it, while those in group (2) learn an aversion and subsequently avoid consuming sucrose [17]. 

The initial raw data from microarray was processed by using GeneSpring software (GX 7.3) and normalization performed using a perchip 75th percentile method, which allows for comparison among chips. Three experimental comparisons were made including conditioned versus control, pseudoconditioned versus control, and conditioned versus pseudoconditioned. Genes that met our filtering criteria were processed for interactive network pathways by IPA (http://www.ingenuity.com) and pathway Studio (PS) (http://www.ariadnegenomics.com/products/pathway-studio/). Although PS software can perform microarray raw data normalization, for the uniformity across different networking software, we used processed data. The processed data was uploaded to IPA and PS with the Agilent IDs, fold values (observation1), and *P* values (observation1) for building networks and pathways. Details for uploading data step by step for IPA and PS can be found in [18].

## 3. Results

### 3.1. CTA Inducse Differential Expression of Many Unique Genes in All Three Groups

**Figure 1:**
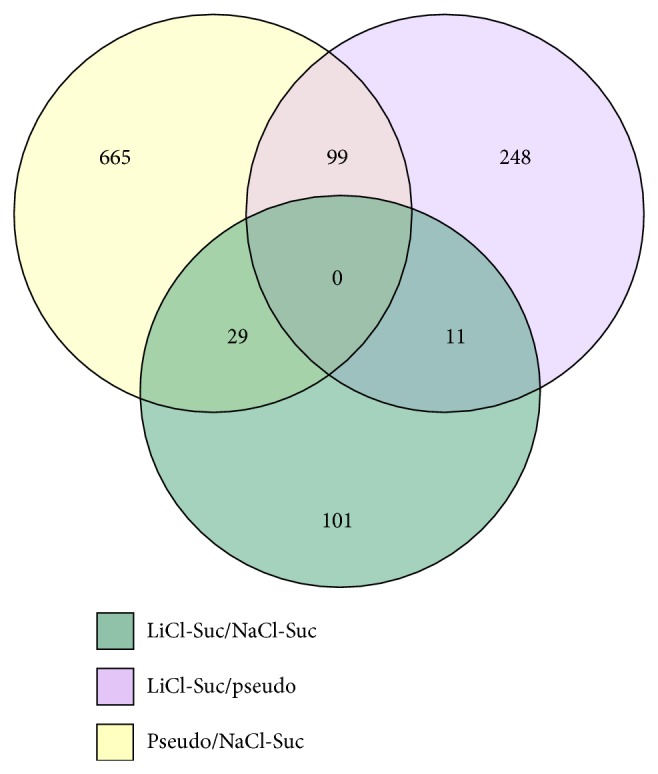
Venn diagram of differentially expressed genes from microarray analysis. The model represents the comparison between the overlapping map of the number of genes in CTA animal groups. The overview is depicted with circles, green circle: LiCl-sucrose/NaCl-sucrose conditioning, purple: LiCl-sucrose/pseudoconditioning, and yellow: pseudoconditioning with NaCl and-Sucrose. The genes selected from each group are with cut-off limits of *P* values ≤0.05 and fold values ≥2.

The oligo-nucleotide microarray of amygdala tissue showed 248 genes differentially regulated in conditioned versus pseudoconditioned, 101 genes in conditioned versus control, and 665 genes in pseudoconditioned versus control groups with *P* values ≤0.05 and fold values ≥2. IPA analyses revealed a lack of commonly regulated genes among all three groups (Figure 1). However, eleven genes were commonly regulated between conditioned versus pseudoconditioned and conditioned versus control, twenty-nine genes between conditioned versus control and pseudoconditioned versus control, and another ninety-nine between conditioned versus pseudoconditioned and pseudoconditioned versus control (Figure 1). 

### 3.2. IPA Reveals Regulation of Many Unique Pathways

**Figure 2:**
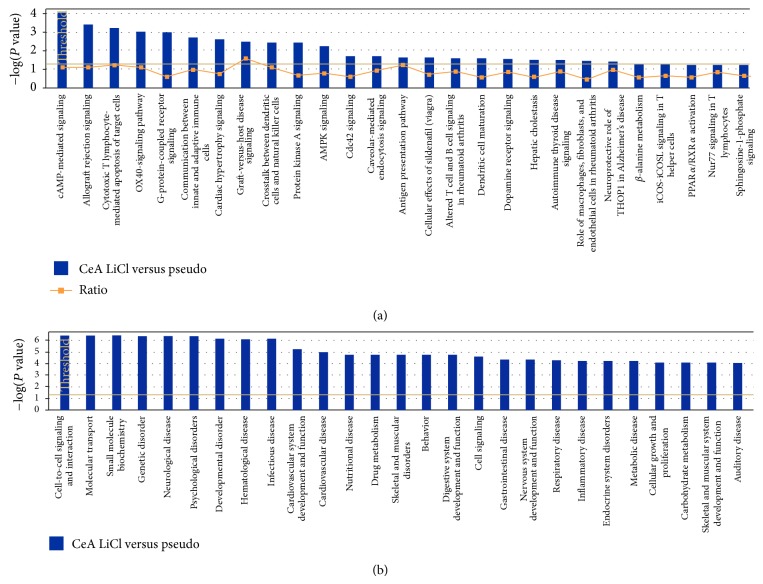
Top networks affected by differentially expressed genes in conditioned group over pseudoconditioned group. (a) Top canonical pathways affected by conditioned group. The −log (*P* values) shown on the *y*-axis are the *P* values obtained by IPA software from the number of genes from the microarray data with the cut-off parameters to the total number of genes present in the particular pathway. (b) Top biofunctional networks affected by conditioned group. The −log (*P* values) shown on the *y*-axis are the *P* values obtained by IPA software from the number of genes from the microarray data with the cut-off parameters to the total number of genes present in the particular pathway.

**Figure 3:**
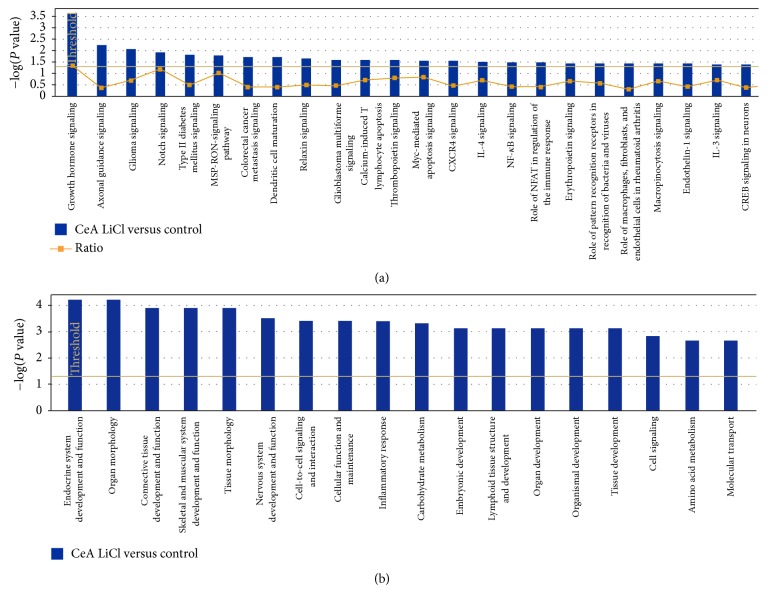
Top networks affected by differentially expressed genes in conditioned group over saline group. (a) Top canonical pathways affected by conditioned group. (b) Top biofunctional networks affected by conditioned group. The −log (*P* values) shown on the *y*-axis are the *P* values obtained by IPA software from the number of genes from the microarray data with the cut-off parameters to the total number of genes present in the particular pathway.

**Figure 4:**
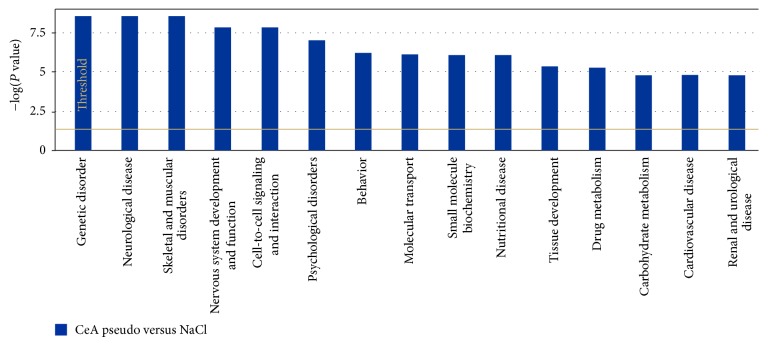
Top networks affected by differentially expressed genes in pseudoconditioned group over saline group. Top canonical pathways affected by pseudoconditioned group. The −log (*P* values) shown on the *y*-axis are the *P* values obtained by IPA software from the number of genes from the microarray data with the cut-off parameters to the total number of genes present in the particular pathway.

In order to understand the interaction between different genes, we generated canonical pathways using IPA software. Based on the input information, IPA generates canonical pathways involving these genes with the *P* value (occurrence) and ratio (frequency). Though the IPA analysis showed few commonly regulated canonical pathways in all three groups, there are many unique canonical pathways regulated within each group. The top canonical pathways regulated in conditioned versus pseudoconditioned were cAMP-mediated signaling, allograft rejection signaling, cytotoxic T-lymphocyte signaling, and G-protein-coupled receptor signaling (Figure 2(a)). The top biofunctional networks in this comparison were cell-to-cell signaling, molecular transport, genetic disorders, neurological diseases, psychological disorders, and developmental disorders (Figure 2(b)). The top canonical pathways regulated in amygdala of conditioned versus control were growth hormone signaling, axon guidance signaling, Notch signaling, type II diabetes signaling, and other immune-related signaling pathways such as chemokine networks, NF*κ*B signaling and IL-4 signaling (Figure 3(a)). The top biofunctions in this comparison were endocrine system development and function, organ morphology, connective tissue development and function, skeletal muscle system development, and nervous system development and function (Figure 3(b)). Finally, the top-canonical pathways regulated in amygdala of pseudoconditioned versus control were G-protein coupled receptor signaling, cAMP-mediated signaling, TR/RXR activation, neuropathic pain signaling, CRH/CRF signaling, and other receptor signaling pathways (Figure 4). The top biofunctional networks for this comparison were genetic disorders, neurological disorders, skeletal and muscular disorders, psychological disorders, and behavioral disorders. Although we observed more genes differentially regulated in pseudoconditioned versus control compared to other group comparisons, fewer canonical pathways were revealed.

### 3.3. Amygdala Plays an Important Role in CTA via Regulation of Many Pathways

**Figure 5:**
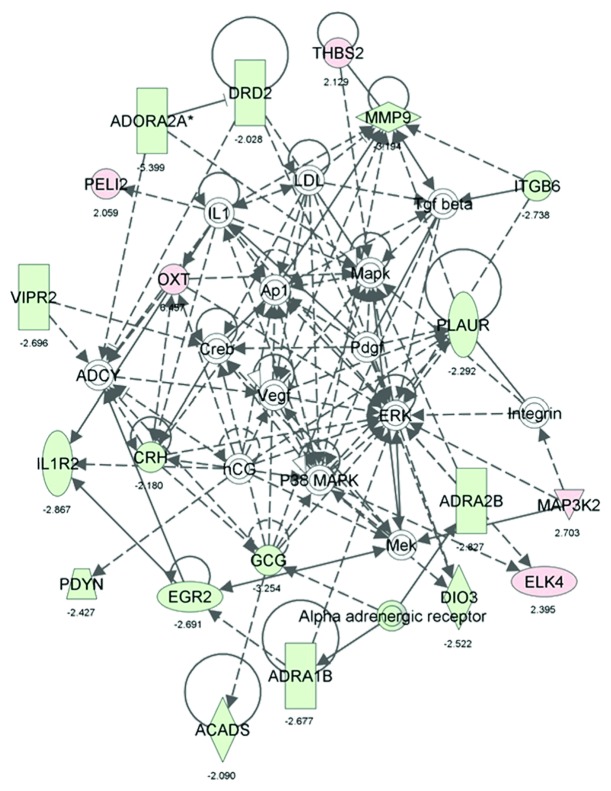
Network of genes involved in nervous systems development and function. Differentially expressed genes from conditioned group over pseudoconditioned group with the selected cut-off *P* values and fold values were used to generate interacting networks from IPA software. The genes shown in green are downregulated in microarray data and those shown in red were upregulated. The intensity of color is in proportion to the fold values. Genes or molecules without any color are intermediate in the network but not found in our microarray data. The indirect relations between the genes were shown in dotted or solid arrows which represent direct interaction. The values below the genes shown here are the fold values.

**Figure 6:**
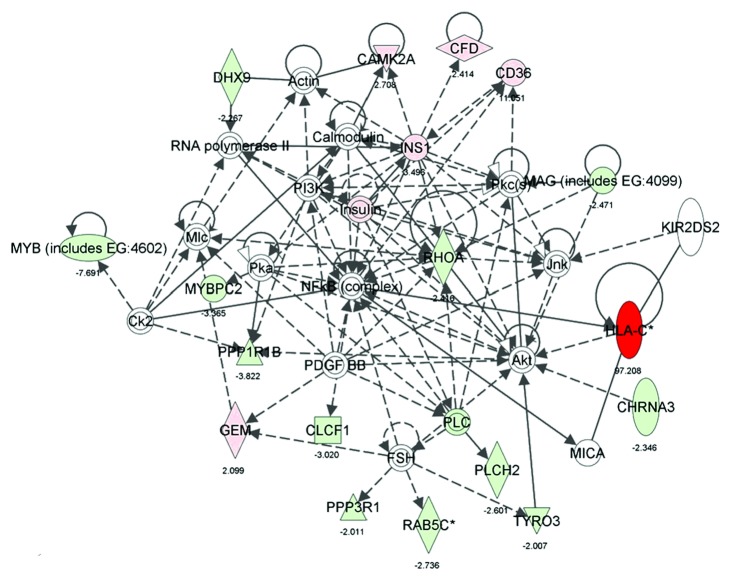
Network of genes involved in cell-to-cell signaling and interaction. Differentially expressed genes from conditioned group over pseudoconditioned group with the selected cut-off *P* values and fold values were used to generate interacting networks from IPA software. The genes shown in green are downregulated in microarray data and those shown in red were upregulated. The intensity of color is in proportionate with the fold values. Genes or molecules without any color are the intermediate in the network but not found in our microarray data. The indirect relations between the genes were shown in dotted arrows and solid arrows represent direct interaction. The values below the genes shown here are the fold values.

We also generated common networks based on the connectivity of these genes using IPA and PS software. Based on the input information, the genes that are downregulated are shown in green and the upregulated genes in red. The differentially expressed genes in amygdala of conditioned groups such as dopamine receptor D2 (-2), MMP9 (-3), adrenergic receptor (-2.8), oxytocin (6.4), corticotropin releasing hormone or CRH (-2), IL-2 receptor (-2.8), VIPR2 (-2.8), mitogen-activated protein kinase kinase kinase 2 or MAP3K (2.7), glucagon (-3.2), EGR2 (-2.6), calcium/calmodulin-dependent protein kinase II or CAMKIIA (2.7), insulin 1 (3.5), cholinergic receptor, nicotinic (-2.3), glycine receptor alpha 2 (2.2), and some sodium channel were used to generate networks and pathways using IPA. Many of the genes are involved in regulation of cellular pathways such as Erk signaling, Creb signaling, Mapk signaling, adrenergic receptor signaling (Figure 5), insulin, PI3K, NFkB, Akt, and CamK signaling (Figure 6), glutamic acid metabolism, calcium signaling, glutamate receptor signaling and dopamine receptor signaling. 

**Figure 7:**
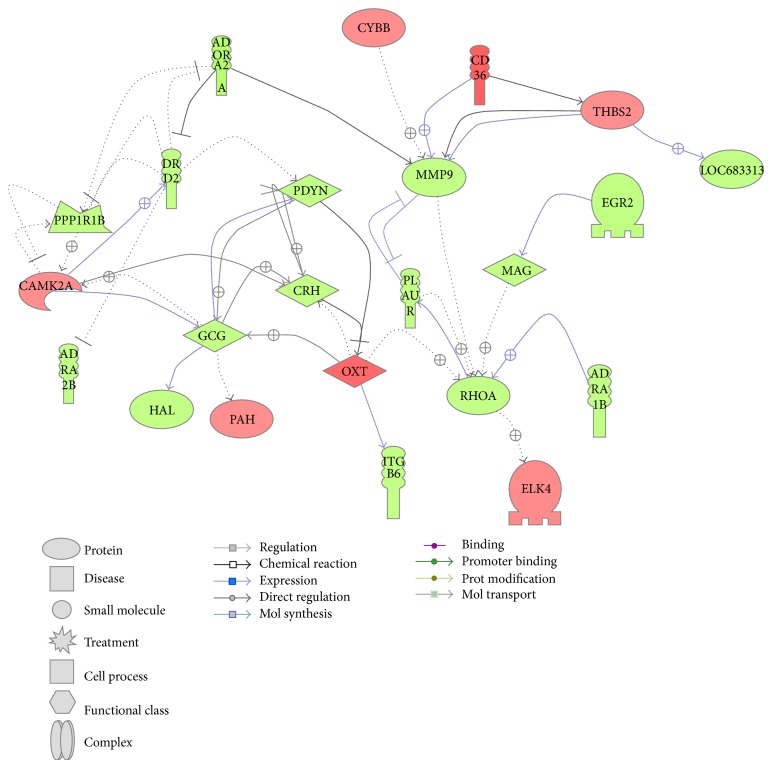
Schematic diagram of network of genes involved in protein and transcriptional regulation network. Differentially expressed genes from conditioned group over pseudoconditioned group with the selected cut-off *P* values and fold values were used to generate interacting networks from Pathway Studio software. The genes shown in green are downregulated in microarray data and those shown in red were upregulated. The indirect interactions between the genes were shown in dotted arrows and solid arrows represent direct interaction.

**Figure 8:**
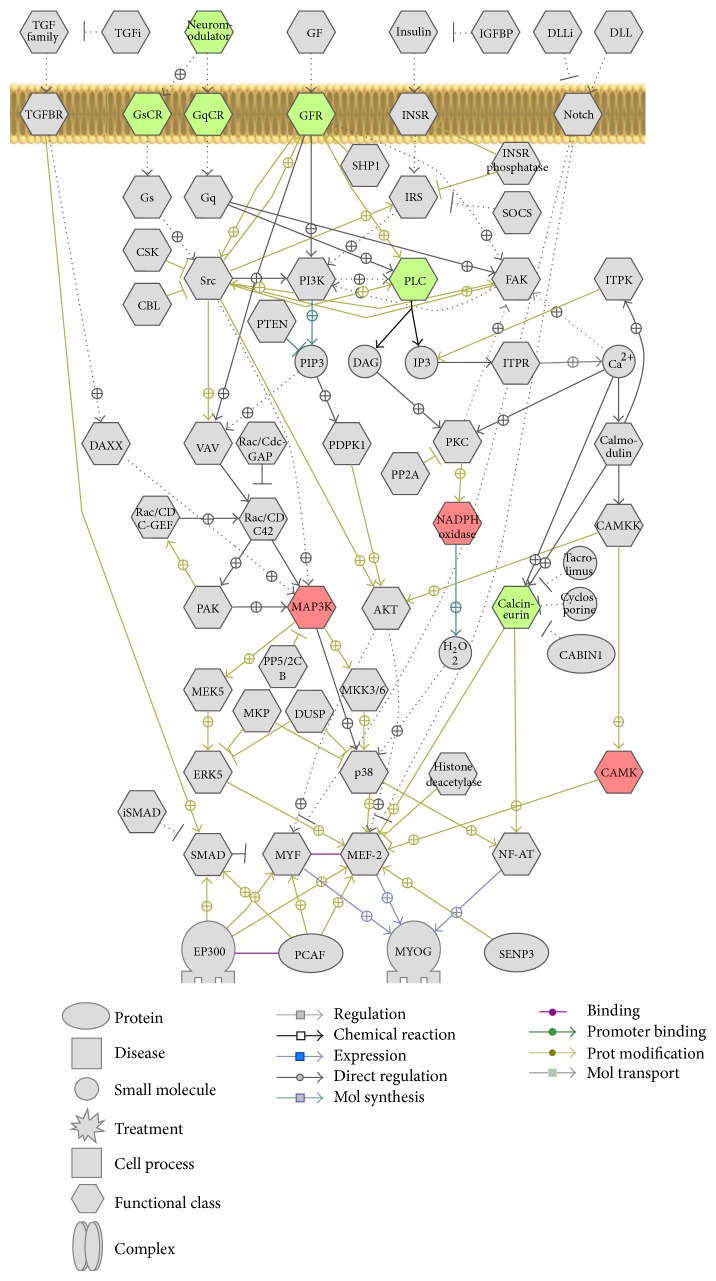
Schematic diagram of network of genes involved in myogenesis and control network. Differentially expressed genes from conditioned group over pseudoconditioned group with the selected cut-off *P* values and fold values were used to generate interacting networks from Pathway Studio software. The genes shown in green are downregulated in microarray data and those shown in red were upregulated. The indirect interactions between the genes were shown in dotted arrows and solid arrows represent direct interaction.

Pathway Studio revealed that the greatest number of genes was represented in networks associated with gonadotropin cell activation, myogenesis control, transcriptional regulation and protein regulation. Several hormones (CRH, glucagon, and oxytocin), receptors (DRD2, ADRA1B, ADRA2A, and ADRA2B), and kinases (CamK2A) function in protein and transcriptional regulation networks (Figure 7). G-protein coupled receptors (GqCR) and kinases such as MAPK and CAMK function in myogenesis networks (Figure 8).

## 4. Discussion

In the present report we utilized our previously published experimental data to identify the gene network pathways in conditioned taste aversion (CTA). Our previous study [17] clearly establishes the link between CTA and amygdala. Although we demonstrated that the differentially expressed genes in amygdala of CTA-learned animal are specific to the taste behavior and blocked central insulin signaling can delay CTA learning in our previous study, characterizing the role(s) of other genes in behavior is very time consuming and expensive. Therefore, as a first step to understand the role of these differentially regulated genes in amygdala of CTA animal, we explored various bioinformatics tools. Therefore, the analysis presented in this study will provide an in-depth view in deciphering the pathways that are likely to be playing a major role in taste guided behavior. We identify the key gene regulatory pathways that are altered in the CTA behavior groups. The molecular links provided are important for mechanistic understanding in the field of gustatory behavior.

Identifying the target molecules and their regulatory networks is the first step for dealing with large sets of gene array data. As CTA process involves ingestion of sucrose solution and treatment with LiCl, there is a possibility of identifying genes that are induced by orosensory signals (sucrose), visceral signals (LiCl), oromotor signals (i.e., continued sucrose intake in control versus avoidance in CTA animals), and/or learning. Understanding offered by evaluating these important regions identifies the genes regulated in learning. Furthermore we compared gene expression between three different groups of animals we reasoned that differentially regulated genes in CTA versus pseudoconditioned animals would yield genes associated with CTA learning as well as differences in orosensory signals induced by sucrose intake. This is because both groups received equal treatment with LiCl with the exception that pseudoconditioned animals received noncontingent pairing of sucrose and LiCl and, thus, did not learn a CTA and continued to consume sucrose. This approach clearly identifies that effect of sucrose consumption on behavior and therefore processes occurring at the molecular level identified under these conditions will be useful for mechanistic understanding. Comparison of the differentially regulated genes between CTA with control animals would yield both learning-related genes as well as those associated with LiCl treatment and differences in orosensory signals induced by sucrose intake. 

### 4.1. CTA Downregulates Key Pathways in the Brain

Differentially regulated genes in CTA versus pseudoconditioned animals (Figure 5) revealed that most genes in the amygdala were downregulated. The selected genes that are differentially regulated in CTA group compared to pseudoconditioned were also tested in visual cortex, a brain region unrelated to taste circuit [22] by qRT-PCR. Our qRT-PCR data showed that glucagon, dopamine receptor 2, major histocompatibility complex, class I, C (HLA-C), glycine receptor, alpha subunit 2, insulin 1, and oxytocin did not show any significant change in visual cortex further confirming their specific role in CTA learning [17]. One of the key genes upregulated was oxytocin which is known to be induced by conditions of hyperosmolarity. Oxytocin-containing parvocellular neurons of the hypothalamic paraventricular nucleus send their projections to brain regions involved in ingestive behavior thereby inhibiting intake of osmoles in food and water [23–25]. In the context of learning oxytocin has been shown to enhance retention of CTA memory in chicken when administered intracerebrally [26]. Evidence further supports a role for oxytocin in behavioral response to anorexic agents such as cholecystokinin (CCK) through the activation of CCK receptor alpha [27]. More interestingly, the oxytocin was found to decrease the expression of CRH, which is downregulated in our microarray data [17]. As we know that CRH is involved in stress and taste preference [28–30], it could be possible that the stress releasing hormone oxytocin was induced to regulate the expression of CRH [31], which is downregulated in our microarray data. The Pathway Studio analysis (Figure 7) also showed that CRH has a direct effect on oxytocin as a response to stress and oxytocin directly regulates glucagon. From these observations it was evident that oxytocin plays an important role in regulating pathways involved in food and liquid intake, memory retention, and osmolarity in amygdala during CTA learning.

Cyclic-AMP Response Element Binding (CREB) protein plays an important role in learning and long-term memory (LTM) formation, which is comprised of a family of transcriptional factors that regulate transcription of genes by binding cAMP response element [32]. The phosphorylation of these CREB proteins is a key regulator of its activation or inhibition by various kinases. Previous reports also suggest that vasoactive intestinal peptide receptor 2 (VIPR2) can increase the phosphorylation of CREB, which is downregulated in our microarray data. Although VIPR2 downregulation in our data suggests the reduced activation of CREB, its effect may be compensated by other CREB activators such as insulin, MAPK, and CamKII [32, 33] which are significantly upregulated in our data. The IPA generated network of genes also suggests that MAP3K is involved in activation of ERK via activation of Erk kinase (Mek), which in turn is increased by oxytocin (Figure 5). The myogenesis control pathway from Pathway Studio also suggests the interaction of MAP3K with other kinases such as CAMK and neurotransmitter calcineurin (Figure 8). This in turn suggests that many of these kinases have direct or indirect effect on many transcriptional factors like CREB and MEF, which regulates multiple pathways including learning, memory, and myogenesis.

Our microarray analysis of amygdala also shows the downregulation of early growth response gene (EGR-2), which is a family of transcriptional factors involved in many cellular pathways. The recent studies on EGR2 knockout mice showed that deletion of this gene in mice does not impair any CTA learning and memory; in fact these knockout mice were found to have improved motor learning and object recognition memory [34]. Earlier reports also suggest that the EGR2 is one of the downstream targets of CREB transcriptional factor [35]. The downregulation of this gene in the amygdala of long-term CTA in mice suggests its possible role in developing object recognition memory and motor learning as part of the aversion learning paradigm. 

The involvement of noradrenergic system in the memory retention has been noted previously [36, 37]. Adrenergic receptor antagonists are found to induce activity of noradrenergic neurons by blocking autoreceptors [38]. Recent studies also showed that an *α*2-adrenergic blocker, yohimbine (1 mg/kg) suppressed the CTA acquisition and retention in rats [39]. In another study, an alpha-2 adrenoreceptor antagonist, methoxy-idazoxan (RX821002), was found to enhance the extinction of CTA in rats by enhancing arousal, alertness, awareness, or attention [36]. Also the adrenergic receptor alpha-1B protein was found to involve in expression of EGR-2, which is also downregulated in our data [40]. Based on our IPA analysis we suggest that EGR-2 is involved in expression of IL-1 receptor, which is also downregulated in the microarray data (Figure 5). In addition to these functions, adrenergic receptors are known to control blood glucose homeostasis through hormonal and neuronal actions which influence both glucose production and glucose disposal. Among them *α*2 adrenergic receptors were found to inhibit insulin levels and increase glucagon secretions [41, 42]. Recent studies in *α*_2_-Adrenoceptors knockout mice showed lower levels of blood glucose, higher insulin in plasma when compared to wild type [43]. The increasing level of glucagon was also reported to induce hepatic glucose production by phosphorylation of transcription factor CREB at Ser133 [44]. The authors also suggest that the elevated levels of glucagon and increasing insulin resistance result in nonsuppressible hepatic glucose production in type 2 diabetes patients. The IPA analysis also suggests that glucagon can be induced by CRH. In support of these findings we observed downregulation of glucagon, CRH, adrenergic receptor alpha 2b and 1b, and EGR-2 in our microarray data (http://www.ebi.ac.uk/microarray-as/ae/; accession number E-MEXP-3029). Pathway Studio also showed direct or indirect relations between adrenergic receptors, dopamine receptors, CRH, MMP9, EGR2, and oxytocin (Figure 7). Though the actual role of adrenergic receptors in memory retention is not yet understood, the down regulation of these receptors may be critical to regulate other important genes such as EGR-2, glucagon, and CRH which are directly involved in ingestive behavior. 

The microarray analysis of amygdala in CTA-learned animal also showed the downregulation of a protooncogene family protein casitas B-lineage lymphoma-like 1 (c-Cbl) by 92.3 fold (Array Express; E-MEXP-3029). This protein contains multiple domains including a RING-finger domain and ubiquitin-associated domain. The Cbl family proteins recruit ubiquitin-conjugate enzymes to RING-finger domain and add ubiquitin to the target proteins. Ubiquitination of target protein can either activate signal transduction or target protein degradation. The expression levels of these cbl family genes were found to be expressed in many tissues including brain [45]. To understand the role of this protein in neuronal activities including learning and memory, these authors developed Cbl-b null mice. These null mice were found to have normal motor coordination and learning in the Morris water-maze task but enhanced retention of long-term memory. These results suggest that Cbl-b is a negative regulator in long-term memory. The authors also suggest that glutamatergic transmission is facilitated in these Cbl null mice and may contribute to the enhanced long-term memory retention found in Cbl-b null mice. From these observations it was evident that the downregulation of c-Cbl in amygdala may favor enhanced long-term memory, which is essential for CTA learning.

In addition to these we also find the downregulation of an important metalloprotease, MMP-9 by 3.2-fold. Matrix metalloproteinases (MMPs) are family of extracellular matrix-degrading enzymes which are required for the degradation of extracellular matrix [46, 47]. Among these MMPs MMP9 (gelatinase B) is found to be very important for synaptic plasticity, learning, and memory [48]. These authors also showed that *β*-dystroglycan is a synaptic target for MMP-9. In a separate experiment these MMP-9 levels were found to be decreased in hippocampus of cocaine abusers [49]. IPA and PS also showed that MMP9 interacts with many genes in the networks shown in Figures 5 and 7 directly or indirectly. These authors suggest the downregulation of these genes involved in extracellular matrix remodeling and thereby favoring synaptic plasticity. The downregulation of this gene in microarray suggests that CTA induces plasticity via regulation of these extracellular matrixes.

### 4.2. CTA Affects Synaptic Plasticity, Food Intake, and Long-Term Memory Formation in Amygdala by Induction of Insulin-1, MHC-I, and Glycine Receptor

Insulin, a peptide hormone, is a very important hormone for food and energy balance. In addition to its role in energy balance, insulin and its receptors are found to involve in control of body weight and reproduction, learning and memory, axon path finding, and ethanol response [50–53]. The recent findings in *Drosophila* indicated that insulin-like receptor signaling systems are essential for the dynamic regulation of noxious food intake according to the animal's energy state [54]. Also insulin is found to stimulate both CREB phosphorylation and transcriptional activation in HepG2 and 3T3-L1 cell lines, which further demonstrate the insulin regulation of CREB [33]. IPA analysis also suggested that CD36 (which is also upregulated by 11-fold) can induce the expression of insulin (Figure 6). The pathway also showed the activation of CAMKIIA, an important kinase that activates CREB, by insulin. Our microarray data also showed the upregulation of this kinase by 2.7-fold (ArrayExpress; E-MEXP-3029). The overexpression of insulin in the amygdala of CTA animal suggests its potential role in modulating energy balance, food intake and long-term memory formation. 

Major histocompatibility complex class I (MHC I) molecules are the proteins that are important for immune response to foreign molecules/antigens. These MHC I molecules were found to be the downstream targets of transcriptional factor CREB, required for synaptic plasticity. The expression of these immune complexes was also evident in neurons that undergo activity-dependent, long-term structural and synaptic modifications [55]. The authors also identified that these MHC I expression are very essential for proper development of central nervous systems otherwise the genetically deficient mice for MHC I found to have enhanced NMDA-receptor-dependent long-term potentiation (LTP) and absence of long-term depression (LTD) in hippocampus of adult mice. These mutant mice were also found to have altered synaptic plasticity. The recent experiments in their laboratory suggest the dual role for MHC I in homeostatic plasticity which in turn may set limits on the magnitude and direction of Hebbian synaptic plasticity [56]. The IPA software showed that this MHC I interacts with NF*κ*B-signaling pathway, which is also one of the important immune-related network (Figure 6). The microarray analysis of amygdala of CTA-learned animal in the current study showed upregulation of this gene by 97-fold. From these observations, it can be evident that CTA may induce plasticity by regulation of MHC I proteins in amygdala.

## 5. Conclusions

In this study we have identified that the conditional taste aversion paradigm is regulated through three major pathways, namely, neuronal plasticity, cognition and memory, and finally food and liquid intake. The neuronal plasticity may be manipulated by the regulation of immune complexes such as MHC-I and other extracellular matrix proteases such as MMP-9. Then the neuronal plasticity enables the cognition and memory of the ingested food, which in turn manipulates the animal's food and liquid intake. cAMP response element-binding protein may be acting as a major molecule in manipulating cognition and memory through the regulation of various kinases such as CamKII and Map3K. The CREB may be further regulated by hormones such as oxytocin, insulin and glucagon in CTA mechanism. The downstream targets of CREB such as EGR2 and IL-1 may also participate in regulation of memory formation. Also we observed the regulation of receptors such as glycine receptor, adrenergic receptor, and VIP receptors which may further regulate memory formation via regulation of CREB. As a consequence of stress induced by noxious agents (LiCl), the stress releasing peptides such as oxytocin may be involving the regulation of stress induced peptides like CRH. In addition to these events some other binding proteins such as cCbl may be involved in improving animal's long-term memory required for CTA learning.

## References

[1] Garcia J., Kimeldorf D. J., Koelling R. A. (1955). Conditioned aversion to saccharin resulting from exposure to gamma radiation. *Science*.

[2] Yamamoto T., Fujimoto Y., Shimura T., Sakai N. (1995). Conditioned taste aversion in rats with excitotoxic brain lesions. *Neuroscience Research*.

[3] Yasoshima Y., Morimoto T., Yamamoto T. (2000). Different disruptive effects on the acquisition and expression of conditioned taste aversion by blockades of amygdalar ionotropic and metabotropic glutamatergic receptor subtypes in rats. *Brain Research*.

[4] Touzani K., Sclafani A. (2001). Conditioned flavor preference and aversion: role of the lateral hypothalamus. *Behavioral Neuroscience*.

[5] Andre J. S., Reilly S. (2007). Effects of central and basolateral amygdala lesions on conditioned taste aversion and latent inhibition. *Behavioral Neuroscience*.

[6] Grossman S. E., Fontanini A., Wieskopf J. S., Katz D. B. (2008). Learning-related plasticity of temporal coding in simultaneously recorded amygdala-cortical ensembles. *Journal of Neuroscience*.

[7] Lamprecht R., Dudai Y. (1995). Differential modulation of brain immediate early genes by intraperitoneal LiCl. *NeuroReport*.

[8] Lamprecht R., Hazvi S., Dudai Y. (1997). cAMP response element-binding protein in the amygdala is required for long- but not short-term conditioned taste aversion memory. *Journal of Neuroscience*.

[9] Yasoshima Y., Scott T. R., Yamamoto T. (2006). Memory-dependent c-Fos expression in the nucleus accumbens and extended amygdala following the expression of a conditioned taste aversive in the rat. *Neuroscience*.

[10] Galaverna O. G., Seeley R. J., Berridge K. C., Grill H. J., Epstein A. N., Schulkin J. (1993). Lesions of the central nucleus of the amygdala I: effects on taste reactivity, taste aversion learning and sodium appetite. *Behavioural Brain Research*.

[11] Gallo M., Roldan G., Bures J. (1992). Differential involvement of gustatory insular cortex and amygdala in the acquisition and retrieval of conditioned taste aversion in rats. *Behavioural Brain Research*.

[12] Roldan G., Bures J. (1994). Tetrodotoxin blockade of amygdala overlapping with poisoning impairs acquisition of conditioned taste aversion in rats. *Behavioural Brain Research*.

[13] Lamprecht R., Dudai Y. (1996). Transient expression of c-Fos in rat amygdala during training is required for encoding conditioned taste aversion memory. *Learning Memory*.

[14] Yasoshima Y., Sako N., Senba E., Yamamoto T. (2006). Acute suppression, but not chronic genetic deficiency, of c-fos gene expression impairs long-term memory in aversive taste learning. *Proceedings of the National Academy of Sciences of the United States of America*.

[15] Koh M. T., Thiele T. E., Bernstein I. L. (2002). Inhibition of protein kinase A activity interferes with long-term, but not short-term, memory of conditioned taste aversions. *Behavioral Neuroscience*.

[16] Yasoshima Y., Yamamoto T. (1997). Rat gustatory memory requires protein kinase C activity in the amygdala and cortical gustatory area. *NeuroReport*.

[17] Panguluri S. K., Kuwabara N., Kang Y., Cooper N., Lundy R. F. (2012). Conditioned taste aversion dependent regulation of amygdala gene expression. *Physiology & Behavior*.

[18] Henderson-MacLennan N. K., Papp J. C., Talbot C. C., McCabe E. R. B., Presson A. P. (2010). Pathway analysis software: annotation errors and solutions. *Molecular Genetics and Metabolism*.

[19] Mahmoud F. A., Aktas A., Walsh D., Hullihen B. (2011). A pilot study of taste changes among hospice inpatients with advanced cancer. *American Journal of Hospice and Palliative Medicine*.

[20] Berteretche M. V., Dalix A. M., D'Ornano A. M. C., Bellisle F., Khayat D., Faurion A. (2004). Decreased taste sensitivity in cancer patients under chemotherapy. *Supportive Care in Cancer*.

[21] Marfaing-Jallat P., Portha B., Pénicaud L. (1995). Altered conditioned taste aversion and glucose utilization in related brain nuclei of diabetic GK rats. *Brain Research Bulletin*.

[22] Krivanek J. (2001). Conditioned taste aversion and Ca/calmodulin-dependent kinase II in the parabrachial nucleus of rats. *Neurobiology of Learning and Memory*.

[23] Rinaman L., Vollmer R. R., Karam J., Phillips D., Li X., Amico J. A. (2005). Dehydration anorexia is attenuated in oxytocin-deficient mice. *American Journal of Physiology—Regulatory Integrative and Comparative Physiology*.

[24] Sawchenko P. E., Swanson L. W. (1982). Immunohistochemical identification of neurons in the paraventricular nucleus of the hypothalamus that project to the medulla or to the spinal cord in the rat. *Journal of Comparative Neurology*.

[25] Puryear R., Rigatto K. V., Amico J. A., Morris M. (2001). Enhanced salt intake in oxytocin deficient mice. *Experimental Neurology*.

[26] Davis J. L., Pico R. M., Cherkin A. (1983). Dose-dependent and time-dependent action of oxytocin on chick memory. *Brain Research*.

[27] Onaka T., Yagi K. (1998). Oxytocin release from the neurohypophysis after the taste stimuli previously paired with intravenous cholecystokinin in anaesthetized rats. *Journal of Neuroendocrinology*.

[28] Heinrichs S. C., Klaassen A., Koob G. F., Schulteis G., Ahmed S., de Souza E. B. (1998). Corticotropin-releasing factor receptor blockade enhances conditioned aversive properties of cocaine in rats. *Psychopharmacology*.

[29] Heinrichs S. C., Richard D. (1999). The role of corticotropin-releasing factor and urocortin in the modulation of ingestive behavior. *Neuropeptides*.

[30] Inoue K., Valdez G. R., Reyes T. M. (2003). Human urocortin II, a selective agonist for the type 2 corticotropin-releasing factor receptor, decreases feeding and drinking in the rat. *Journal of Pharmacology and Experimental Therapeutics*.

[31] Liu Y., Curtis J. T., Fowler C. D., Spencer C., Houpt T., Wang Z. X. (2001). Differential expression of vasopressin, oxytocin and corticotrophin-releasing hormone messenger RNA in the paraventricular nucleus of the prairie vole brain following stress. *Journal of Neuroendocrinology*.

[32] McClung C. A., Nestler E. J. (2008). Neuroplasticity mediated by altered gene expression. *Neuropsychopharmacology*.

[33] Klemm D. J., Roesler W. J., Boras T., Colton L. A., Felder K., Reusch J. E. B. (1998). Insulin stimulates cAMP-response element binding protein activity in HepG2 and 3T3-L1 cell lines. *Journal of Biological Chemistry*.

[34] Poirier R., Cheval H., Mailhes C., Charnay P., Davis S., Laroche : S. (2007). Paradoxical role of an egr transcription factor family member, egr2/krox20, in learning and memory. *Frontiers in Behavioral Neuroscience*.

[35] Sato K., Kimura T., Ota K. (1995). Changes in plasma vasopressin levels and cardiovascular function due to postural changes in diabetic neuropathy. *The Tohoku Journal of Experimental Medicine*.

[36] Fresquet N., Angst M. J., Schleef C., Gobaille S., Sandner G. (2007). Adrenergic drugs modify the level of noradrenaline in the insular cortex and alter extinction of conditioned taste aversion in rats. *Behavioural Brain Research*.

[37] Liu W., Yuen E. Y., Allen P. B., Feng J., Greengard P., Yan Z. (2006). Adrenergic modulation of NMDA receptors in prefrontal cortex is differentially regulated by RGS proteins and spinophilin. *Proceedings of the National Academy of Sciences of the United States of America*.

[38] Mateo Y., Meana J. J. (1999). Determination of the somatodendritic *α*2-adrenoceptor subtype located in rat locus coeruleus that modulates cortical noradrenaline release in vivo. *European Journal of Pharmacology*.

[39] Ishitobi S., Ayuse T., Yoshida H., Oi K., Toda K., Miyamoto T. (2009). Effects of midazolam on acquisition and extinction of conditioned taste aversion memory in rats. *Neuroscience Letters*.

[40] Herdegen T., Kiessling M., Bele S., Bravo R., Zimmermann M., Gass P. (1993). The KROX-20 transcription factor in the rat central and peripheral nervous systems: novel expression pattern of an immediate early gene-encoded protein. *Neuroscience*.

[41] Hermann L. S., Deckert T. (1977). The effect of epinephrine and isoproterenol on insulin secretion and glucose utilization in isolated islets of Langerhans from mice. *Acta Endocrinologica*.

[42] Filipponi P., Gregorio F., Ferrandina C. (1986). Alpha-adrenergic system in the modulation of pancreatic A and B cell function in normal rats. *Diabetes Research and Clinical Practice*.

[43] Savontaus E., Fagerholm V., Rahkonen O., Scheinin M. (2008). Reduced blood glucose levels, increased insulin levels and improved glucose tolerance in *α*2A-adrenoceptor knockout mice. *European Journal of Pharmacology*.

[44] He L., Sabet A., Djedjos S. (2009). Metformin and insulin suppress hepatic gluconeogenesis through phosphorylation of CREB binding protein. *Cell*.

[45] Tan D. P., Liu Q. Y., Koshiya N., Gu H., Alkon D. (2006). Enhancement of long-term memory retention and short-term synaptic plasticity in cbl-b null mice. *Proceedings of the National Academy of Sciences of the United States of America*.

[46] Yamamoto T., Sasaki G., Sato T., Katayama I., Nishioka K. (1995). Cytokine profile of tumor cells in mycosis fungoides: successful treatment with intra-lesional interferon-*γ* combined with chemotherapy. *Journal of Dermatology*.

[47] Takanami I., Imamuma T., Yamamoto Y., Yamamoto T., Kodaira S. (1995). The rapid transformation of hyperthyroidism to hypothyroidism complicated by myasthenia gravis. *Journal of Thoracic and Cardiovascular Surgery*.

[48] Michaluk P., Kolodziej L., Mioduszewska B. (2007). *β*-Dystroglycan as a target for MMP-9, in response to enhanced neuronal activity. *Journal of Biological Chemistry*.

[49] Mash D. C., Ffrench-Mullen J., Adi N., Qin Y., Buck A., Pablo J. (2007). Gene expression in human hippocampus from cocaine abusers identifies genes which regulate extracellular matrix remodeling. *PLoS One*.

[50] Bruning J. C., Gautam D., Burks D. J. (2000). Role of brain insulin receptor in control of body weight and reproduction. *Science*.

[51] Song J., Wu L., Chen Z., Kohanski R. A., Pick L. (2003). Axons guided by insulin receptor in Drosophila visual system. *Science*.

[52] Moore P. (2003). Controlling how many cells make a fly. *Journal of Biology*.

[53] Kyriaki G. (2003). Brain insulin: regulation, mechanisms of action and functions. *Cellular and Molecular Neurobiology*.

[54] Wu Q., Zhao Z., Shen P. (2005). Regulation of aversion to noxious food by Drosophila neuropeptide Y- and insulin-like systems. *Nature Neuroscience*.

[55] Huh G. S., Boulanger L. M., Du H., Riquelme P. A., Brotz T. M., Shatz C. J. (2000). Functional requirement for class I MHC in CNS development and plasticity. *Science*.

[56] Yamashita M., Yamamoto T. (1995). A case of very slowly progressive high cervical spondylotic myelopathy presenting with symmetric deep sensory deficits in the palms. *Brain and Nerve*.

